# Surgical Brain Metastases: Management and Outcome Related to Prognostic Indexes: A Critical Review of a Ten-Year Series

**DOI:** 10.5402/2011/207103

**Published:** 2011-09-20

**Authors:** Manuela Caroli, Andrea Di Cristofori, Francesca Lucarella, Fabio Angelo Raneri, Francesco Portaluri, Sergio Maria Gaini

**Affiliations:** Department of Neurosurgery, Università degli Studi di Milano, Fondazione IRCCS Ca' Granda-Ospedale Maggiore Policlinico, 20122 Milan, Italy

## Abstract

Brain metastasis are the most common neoplastic lesions of the nervous system. Many cancer patients are diagnosed on the basis of a first clinical presentation of cancer on the basis of a single or multiple brain lesions. Brain metastases are manifestations of primary disease progression and often determine a poor prognosis. Not all patients with a brain metastases undergo surgery: many are submitted to alternative or palliative treatments. Management of patients with brain metastases is still controversial, and many studies have been developed to determine which is the best therapy. Furthermore, management of patients operated for a brain metastasis is often difficult. Chemotherapy, stereotactic radiosurgery, panencephalic radiation therapy, and surgery, in combination or alone, are the means most commonly used. We report our experience in the management of a ten-year series of surgical brain metastasis and discuss our results in the preoperative and postoperative management of this complex condition.

## 1. Introduction

Brain metastases represent the most frequent type of intracranial tumors, being a common complication of cancer. The most common sources of brain metastases are lung, breast, or melanoma, and in as many as 15% of patients, primitive localization remains uncertain [1]. The frequency of brain metastases has increased over time, probably as a result of advances in neuroimaging procedures and improvements in the treatment of primary and systemic cancer disease. Though nowadays, head CT studies and MRIs offer high quality imaging, there are not characteristic features which enable us to distinguish brain metastases from primary malignant brain tumors or nonneoplastic conditions [1, 2]. Tissue biopsy is necessary in patients with an unidentified primary tumor before radiotherapy and/or chemotherapy. Most patients who develop brain metastases have a relatively short prognosis even if initial treatment is often successful. Survival is determined by the progression of systemic disease or by ineffective control of neurological disease [3, 4].

Several factors are important for determining prognosis and have been combined to determine survival scores: a high Karnofsky performance Status, a single brain metastasis, absence of further systemic metastases, satisfactory control of primary tumor and younger age at diagnosis. Prognosis has been determined according to these factors, among others, in the recursive partitioning analysis (RPA) classification [5].

Treatment options in patients with newly diagnosed brain metastases have improved over the years, and the choice of therapy should take into account patient conditions, number, size, and histology of metastases. The combination of surgery and whole-brain radiotherapy (WBRT) is superior to WBRT alone for the treatment of single brain lesions in patients with good prognostic scores [6]. A total surgical exeresis can resolve acute problems due to increased intracranial pressure and irritative and focal neurological deficits. A valid alternative can be radiosurgery, with or without WBRT, if lesion diameter is under 3 cm. Moreover, radiosurgery enables treatment surgically inaccessible metastases [7].

## 2. Prognostic Factors

### 2.1. Histology

Histology of primary tumor is a very important prognostic factor. We can find different kind of neoplasia which can be divided according to response against therapies. We propose to define tumors in base of radiosensitivity. Radioresistant tumors are kidney cancer and melanoma [8]. They are considered radioresistant, because they tend to not respond to WBRT. They could be treated by SRS or surgery achieving a good local control [1, 7]. In those tumors, surgery may could be preferred to achieve better local control and a rapid relieve from symptoms, but no evidence can support our opinion. At this time, metastases considered to have worst prognosis as possible develops from lung tumors, renal tumors, and melanoma [8]. 

Moreover, not all tumors have a surgical indication: tumors such as lymphomas or small cells lung carcinoma (SCLC) must not be treated. Particularly, lymphomas are treated with high dosage steroids, whereas SCLC is treated with WBRT. Different authors suggest use of prophylactic brain irradiation in patients affected by SCLC [9]. In these cases, the only reason determining a surgical exeresis is characterized by life-threatening intracranial hypertension.

### 2.2. KPS, RPA, and Other Grading Scales

The most important prognostic factor affecting prognosis is believed to be the Karnofsky performance status (KPS). It has been seen that patients with a low-performance status at diagnosis have a worse outcome than others with better KPS [5, 8]. Another parameter affecting prognosis seems to be the age at diagnosis. 

(i) RPA (recurrence partitioning analysis) relates KPS with age at presentation [5]. Cutoff is fixed at 65 years old. Another parameter taken into account is the absence or presence of disease progression. Obviously, uncontrolled disease relates with poor prognosis [5]. RPA is the most important evaluation scale, at present time, for dividing patients according to prognostic classes. The subdivision into 3 classes makes it simple and easy to use, and it is nowadays commonly used to assess surgical eligibility for patients. In addition, good RPA often indicates a better response and compliance to adjuvant therapies, meaning a relative good outcome in surgically treated patients. On the contrary, low RPA indicates a progression of the primary disease and a probable worse response to adjuvant therapies with a consequent worse surgical outcome. RPA is useful to candidate to surgery asymptomatic or low-symptomatic patients, but, in our opinion, it has some characteristics that do not fulfill the clinical needs. Further considerations will be discussed below.

Several other prognostic scales have been described in the literature. In particular, we report the SIR and the GPA grading scores.

(ii) SIR (score index for radiosurgery in brain metastases) is a prognostic score index for patients treated with SRS. It classifies patients into 3 categories according to 5 major prognostic factors: KPS, age at presentation, extracranial disease status, number of brain lesions, and largest brain lesion volume. The SIR has the advantage of including, differently from the RPA, the number of brain metastases, which is now a proven high-significance prognostic factor. In a retrospective study conducted on patients undergoing radiosurgery, SIR demonstrated a significant correlation with survival, showing even a superior accuracy in assessing survival than RPA [10]. Its disadvantage consists of the required calculation of the largest lesion's volume. In fact, this parameter is often assessed only at the time of radiosurgery. Therefore, the SIR seems to miss the point of a prognostic index, which is to predict survival before any treatment decision is made in order to guide the treatment choice itself.

(iii) GPA (graded prognostic assessment) is a prognostic index based on the RTOG (radiation therapy oncology group) database. It is the sum of 3 scores, taking into account 4 prognostic factors: age at presentation, KPS, extracranial metastases, and the number of brain metastases. The GPA, when compared to other prognostic scores, has the advantage of eliminating components that are difficult to quantify (such as primary tumor and extracranial disease control), since they are influenced by diagnostical means. Furthermore, unlike SIR, parameters depending on treatment factors such as the lesion volume are considered. In a recent study, GPA was found to have a prognostic value similar to the RPA and greater than other indices [11].

In Table 1, a brief comparison between the three main prognostic scores is shown.

### 2.3. Extracranial Localizations and Disease Control

Extracranial metastases in systemic cancer relate with a poor prognosis [5]. This is a fundamental factor in treatment choice. Patients at a terminal stage of disease are not eligible for surgery, WBRT or SRS. Only therapy with high dosage of steroids and palliative care can be taken into consideration, as survival in these patients is estimated to be only a few months. Treatment of patients with good performance status and stable extracranial disease can be problematic considering that systemic cancer gives rise to brain metastases and extracranial localizations [12].

## 3. Current Treatment Options

Actual treatment options for brain metastases include surgery, stereotactic radiosurgery (SRS), whole brain radiation therapy (WBRT), and chemotherapy. 

(i) WBRT was the first and, for a long time, elective treatment strategy for brain metastases [13]. Nowadays, its role is controversial, as it has been proven to be associated with high brain toxicity determining severe cognitive impairments, particularly in learning and memory functions [14], and causes a loss in functional autonomy [4]. WBRT remains the treatment of choice for patients with single or multiple brain lesions not selectable for surgery or SRS. On these patients, WBRT results in a median survival period of 3 to 6 months [15]. Differences in dose, timing and fractionation have been studied but do not seem to significantly influence median survival time [16, 17]. In patients submitted to surgery or SRS, the association of WBRT relates to a better local disease control when compared to surgery or stereotactic radiosurgery alone. In addition, WBRT can be used as lifesaving treatment. Anyhow, it has been shown that WBRT fails to improve or to significantly increase global survival rate. Moreover, a gain in survival is associated with worse neurological condition [4]. It is still unclear if WBRT should be used as an adjuvant treatment following surgery or SRS. Kocher and colleagues proposed a close follow-up period after either surgery or SRS with brain MRI, using WBRT as lifesaving treatment [4]. The role of WBRT in patients with a low RPA is still to be established. In a recent study from Komosinska et al., WBRT determined to have more side effects than benefits without improvement of survival rate and quality of life [18]. Randomized studies about long-time survivors may give more accurate information about neurotoxicity from WBRT.

(ii) SRS consists in a single high-dose multiple convergent radiation delivery to a small target volume, minimizing the damage to surrounding brain tissue. It allows treatment of metastases at almost any location in both radiosensitive and radioresistant tumors [7]. According to several authors, SRS determines an inferior local disease control compared to surgery alone [1, 19, 20] though it is less invasive. Nevertheless, it seems to be affected by a similar perioperative mortality rate [1]. Elective indications are small lesions (inferior or equal to 3 cm) without mass effect and with an assumed diagnosis. Important disadvantages are requirement of long-term high-dosage steroids, radionecrosis (needing an additional diagnostical and therapeutical management) and significant patient prostration, with poor possibilities of recovery. SRS can also be used as an adjuvant or a lifesaving treatment. SRS alone is considered to have the same impact on survival rate as surgery followed by SRS. Moreover, SRS combined with WBRT show the same outcome as surgery and WBRT [21].

(iii) Chemotherapy plays an important role for systemic disease control: it is not indicated as a primary therapy for brain metastases [3]. Ewend et al. used Carmustine polymer wafers as local chemotherapy after surgical resection. Each patient also received WBRT; local recurrence rate was extremely low (0%), and survival rate was 33% at 1 year and 25% at 2 years [22]. Furthermore, association of Temozolomide and WBRT has not improved survival rate in several studies [23, 24]. Fotoemustine and Temozolomide have been used as single agents or in combination for the treatment of melanoma metastases with poor results [25–28]. Development of new strategies, such as local pharmacological agents, may give way to new therapeutical options in the near future.

(iv) Surgery is the most invasive treatment compared to the other strategies. On the other hand, it enables definitive histological diagnosis (which is important to set further adjuvant therapies). Moreover, surgical exeresis of the lesion relieves the patient from neurological conditions due to intracranial hypertension (determined by the mass effect of the lesion and the surrounding edema). Contraindications are the presence of uncontrolled disease, very poor prognosis at presentation, and deteriorated clinical conditions. Patients with multiple lesions are often submitted to SRS. Anyhow, Paek et al. reported that patients with 2 or 3 lesions, undergoing exeresis of the dominant lesion and WBRT, showed an equal survival rate as patients with a single lesion [29]. These results were confirmed by Stark and colleagues [30]. Another study performed by Bindal et al. showed better survival rate in patients with resection of all lesions in comparison to patients who underwent exeresis of only dominant lesion [31]. Nevertheless, multiple lesions (>3) are not considered to be surgically treatable [1]. Several additional problems are debated for surgery, in particular the type of exeresis (piecemeal versus en block). In a retrospective study of MD Anderson Cancer Centre, piecemeal resection seemed to increase the risk of dissemination to the leptomeninges (LMD); such risk is higher in posterior fossa lesions after either surgery or SRS [32]. A second study from MD Anderson group demonstrated a higher risk of local recurrence in brain metastasis treated with piecemeal resection than in those treated with en block resection [33]. Surgery followed by WBRT certainly determines a more effective treatment in terms of local and distant recurrence [1, 6]. Contrasting results are reported in studies comparing different treatment options, such as SRS and WBRT versus surgery and WBRT [6]. Therefore, the role of adjuvant therapies after surgical resection is still unclear: there are no clinical studies comparing the results of surgery and/or WBRT versus surgery and SRS. Finally, we lack information concerning patients with multiple metastases, low KPS, and uncontrolled extracranial disease.

## 4. Material and Methods

Patients with cerebral metastases suffer systemic cancer which is often related with a poor prognosis. The neurosurgical procedure is an invasive treatment, and it can be associated with invalidating complications such as hemiplegia or aphasia. The advancing of new and less invasive technologies, particularly SRS, have progressively diminished the role of surgery for the treatment of brain metastases [7]. In our institution, brain metastases are surgically treated according to the following criteria:

(i) necessity of definitive histological diagnosis,

(ii) relatively good prognosis,

(iii) absence of extracranial metastatic localizations,

(iv) life-threatening intracranial hypertension.

In our opinion, these criteria take into account clinical necessities. Patients who did not meet these criteria were submitted to palliative cares. 

Surgical exeresis was performed mostly on single lesions. KPS is a useful parameter for assessing patient's prognosis. However, surgical indication in our series was not strictly based on KPS, for several reasons. First, preoperative KPS, is influenced by reversible pathological conditions, such as brain edema, intracranial hypertension, neurological deficits, or epilepsy. Second, posterior fossa lesions often represent a life-threatening condition, and it is not always easy to overrun such this factor. Third, surgical intervention has become a standard procedure, and complications have diminished through years. Fourth, surgery can relieve or even solve pathological conditions (affecting KPS at presentation) reported above. Last, a histological determination can be assessed. We do not perform surgical procedures in patients with uncontrolled extracranial disease, in patients at high anesthesiological risk, or in patients with more than 3 lesions which are not resectable through one craniotomy. Age is considered a debatable contraindication, since many patients over 65 years of age can sustain general anesthesia. Therefore, we believe that age is indeed a predictive factor on prognosis, but 65 years should not be an absolute cutoff. Furthermore, patients presenting with one to 3 lesions under 3 cm in diameter, without neurological deficits and an assumed histological diagnosis, were sent to SRS, according to scientific evidences [6].

## 5. Results

### 5.1. Our Surgical Series

We report a ten-year series of patients affected by brain metastases who were submitted to surgery. We performed surgical exeresis or biopsy of brain metastasis on 204 patients (128 males and 76 females) admitted at our institution between 2000 and 2009. Surgical exeresis was performed *en block *in all cases. Average age was 59.7 years (range 24–85 years). Twenty-eight patients had more than one lesion, and 8 patients underwent a stereotactic biopsy. We excluded patients submitted to SRS or WBRT before surgery. In 89 cases, a primary tumor diagnosis was known, while in the remaining 115 cases, brain metastases were the first clinical manifestation of cancer. Histology and localizations of lesions are reported in Table 2. Most brain metastases were from lung (99), followed by kidney (22), bowels (16), melanoma (14) and breast (12) cancer. In 34 cases (16.7%), the source of primary tumor remained unknown. All patients were classified according to Gaspar's recursive partitioning analysis (RPA) classes. In our series, 91 patients were in class I, 50 patients in class II and 63 patients in class III. KPS was calculated at admission; average KPS was 79.8. Perioperative death rate was <2%. 

**Figure 1:**
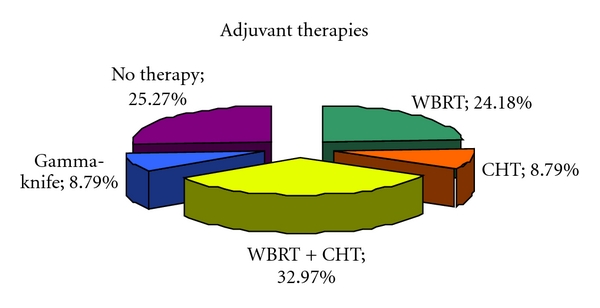
Adjuvant treatments delivered to patients. 82 (33%) patients underwent WBRT plus chemotherapy, 45 (24.2%) patients only WBRT, 19 (8.8%) patients only chemotherapy and 13 (8.79%) patients were sent to SRS or cyber knife. 45 (25.3%) patients have been only followed up or have been sent to palliative cares.

After surgical resection, patients were selected for different further medical treatments in agreement with the oncologists. In particular, 82 patients were submitted to WBRT and chemotherapy, 45 patients to only WBRT, 19 patients were submitted to only chemotherapy, and 13 patients were to SRS or cyber knife. Fourty-five patients had no further treatment or were offered palliative cares. The latter strategy is more often the choice in elderly patients or in patients with extracranial disease (see Figure 1).

**Figure 2:**
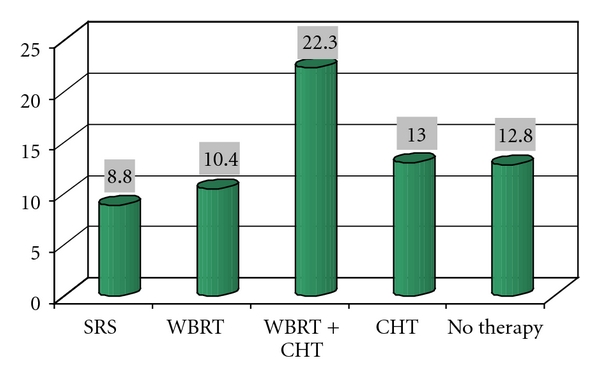
Average survival related to adjuvant therapies.

**Figure 3:**
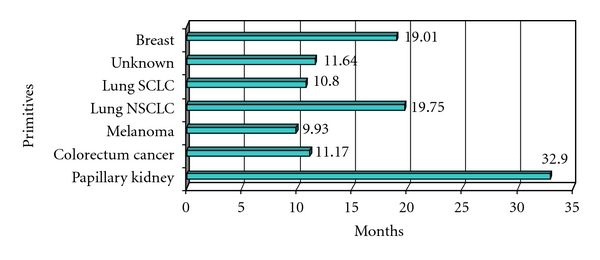
Average survival according to histology.

**Figure 4:**
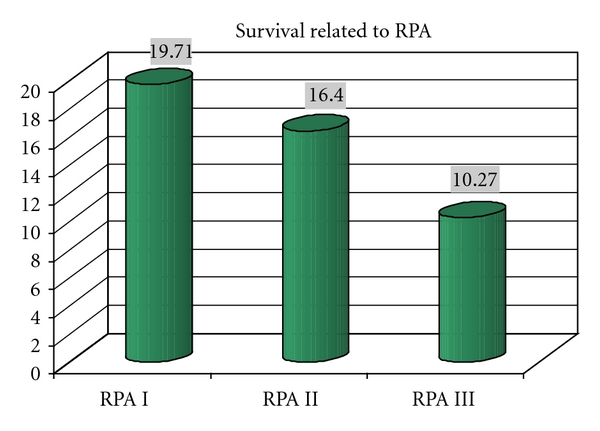
Average survival according to RPA.

We considered followup until November 2010. Median survival was 10 months (range 1–80 months). Survival according to adjuvant therapies is shown in Figure 2. Recurrence rate was 11.27%; 6 patients who did not receive radiotherapy after surgery and with a good disease control underwent a second craniotomy, and only one underwent a third craniotomy. We considered survival rate according to histology and RPA classes (see Tables 3 and 4 and Figures 3 and 4). RPA class I-patients showed a better prognosis than others, with an average survival of 19.7 months (median 10.3 months). This result may be related to a better relief after surgery and consequently to a better compliance to adjuvant treatments. RPA class II-patients had an intermediate prognosis, with an average survival of 16.4 months (median 8.6 months) and RPA class III-patients showed an average survival of 10.3 months (median 3.6 months).

## 6. Discussion

### 6.1. RPA Pitfall

RPA and other grading scales, such as SIR and GPA, take into account mainly KPS, extracranial disease, number of lesions, or lesion's volume. All main grading scales do not take into account patient's neurological conditions. For example, intracranial hypertension due to severe edema determines a rostrocaudal deterioration. This is related to a decreased KPS. Surgical debulking results in an increase of KPS because functional structures (corticospinal tract, Broca or Wernicke's areas) are relieved from compression with an improvement of neurological condition. Moreover, surgical decompression can reverse distress of the ascending reticular activating system, improving a confusional state or coma which were responsible for a low preoperative KPS.

An additional problem is the localization of metastatic lesions: supratentorial lesions present different clinical problems and determine several cerebral dysfunctions. Furthermore, posterior fossa tumors enclose two important problems: they determine life-threatening conditions and they are associated with a high risk of leptomeningeal dissemination (LMD). LMD is considered to have a very poor prognosis (3-4 months) due to the lack of treatment possibilities [34].

Therefore, surgery represents an effective strategy considering not only prognostic factors, but also clinical status. In our opinion, KPS is a good performance status index, but it needs to be reconsidered and adapted according to reversible neurological conditions. Preoperative KPS differs from postoperative KPS: surgery improves neurological deficits, edema, and seizures and leads to a definitive histological diagnosis. Finally, oncological patients can develop a primary brain tumor or another neoplasia. Patchell and collegues have shown that 11% of patients with brain metastases may have nonmetastatic lesions such as an abscess or a primary brain tumor [35].

### 6.2. Which Treatment?

Literature reports different studies concerning the use of surgery, SRS and WBRT. The main debate is upon the benefits and risks of surgery versus radiosurgery. In patients with good RPA (class I or II), indications for SRS and surgery are well defined: SRS is the treatment of choice in patients with a highly probable diagnosis, lesion size <3 cm and without mass effect (<1 cm brain shift) [1]. Patients with intracranial hypertension usually have a low KPS, consequently influencing a low RPA. Such patients would probably take great advantage from intracranial decompression. Therefore, as remarked above, some RPA class III-patients may be treated surgically. Systemic disease control is, in our opinion, an important factor influencing surgical indication. Patients with uncontrolled extracranial disease cannot be eligible for an elective surgical treatment, with the important exception of patients with posterior fossa lesions and clinical signs of cerebellar herniation. Finally, patients with low RPA are commonly treated with WBRT. In a recent study, however, the role of palliative WBRT in RPA class III-patients is debated: according to Komosinska, such patients probably do not benefit from WBRT [18].

### 6.3. Implications of Treatment on Cognitive Functions

The influence of radiation therapies on cognitive functions is a recent matter of study due to the progressive increase in survival and efficacy of treatments. As reported above, SRS and WBRT are related with cognitive impairment [4, 14]. Particularly, radiation therapy is characterized by several adverse reactions (classified as acute, subacute, and delayed) that foreclose the delivery of high-dose X-rays [36, 37]. Such side effects can develop either after WBRT and SRS. Cognitive impairment is a delayed toxic reaction becoming more evident in long survivors [13]. Risk factors related to cognitive impairment are fraction size >2 Gy and whole brain RT. Anyhow, such factors have not been extensively analysed on randomized trials [37]. As possible additional risk factors, we report the cerebral volume irradiated and a long time interval after treatment [37]. Particularly, the necessity to deliver WBRT in the immediate postoperative period still needs to be assessed. Kocher and colleagues proposed to delay brain irradiation preferring a closer radiological followup with short intervals between brain metastases' treatments and the MRI scans and consider WBRT as a terminal option in cases of recurrence or new localizations [4]. Radionecrosis is a further delayed toxic effect of radiations. It involves mainly the white matter, and it develops after both WBRT and, less frequently, SRS [13, 38]. Differential diagnosis is difficult, since no effective diagnostic technique for distinguishing radionecrosis from tumor recurrence is available [39, 40].

### 6.4. Considerations on Survival

The survival data shown are influenced by different clinical parameters which do not offer an effective prediction on prognosis. Particularly, the distinction between class II and class III-patients fails to be completely clear. In fact, class III-patients could have had an intracranial hypertension determining a lower KPS (<70), while class II-patients could have had a good KPS but uncontrolled or only partially controlled metastatic disease, or they could have been more than 65 years old. Considering survival according to histology (Table 3), we noticed that survival rate is strongly related to histological diagnosis, a factor which is not taken into account by RPA or other grading scores. This fact can be obtained comparing average with median survival rate (Tables 3 and 4). Differences may relate with other parameters that are not taken into account considering histology or RPA only. For example, in radioresistant tumors, surgery may be preferred to SRS in order to achieve a better local control and a rapid relief of symptoms even if we have no evidence to support our opinion. Clinical parameters are not taken into account in prognostic indexes such as disease-free interval. As a matter of fact, in our series, many patients were cancer-free for a long term before developing brain metastases, and this finding seems to relate to a better prognosis. Patients developing brain metastases after a long free survival period show a better prognosis than those with a history of rapid development of brain metastases [8, 41, 42]. We also observed a better outcome and survival rate in patients with a long history of neoplastic disease (unpublished data). For this reason, we believe that RPA classes are useful for selecting patients for WBRT but incomplete for assessment of surgical indications. We, therefore, believe RPA analysis may be improved with some adjustments, for example, introducing a risk stratification based on histology, neurological deficits, intracranial hypertension, and long disease-free interval between primary tumor diagnosis and the onset of brain metastasis.

## 7. Conclusions

In conclusion, according to our opinion, surgery still remains the best therapeutical possibility for brain metastases, as it provides a definite histological diagnosis, it relieves epilepsy, and it allows fast debulking of suffering nervous structures, determining an improvement of KPS and a better overall prognosis. These functions cannot be relieved by SRS alone, which remains an effective treatment for lesions without definitive histological diagnosis, poor edema, pharmacologically treated epilepsy, and without neurological deficits. Finally, we believe that more accurate scoring grades should be developed, particularly a grading system taking into account reversible patient's clinical conditions.

## Figures and Tables

**Table:** (a) Recursive partitioning analysis (RPA)

	Class I	Class II	Class III
Age (years)	<65	All patients not in Class I or III	<70
KPS	≥70		
Control of primary tumor	Yes		
Extracranial metastases	No		

**Table:** (b) Score index for radiosurgery in brain metastases (SIR)

	Score 0	Score 1	Score 2
Age (years)	≥60	51–59	<= 50

KPS	≤50	60–70	>70

Systemic disease status	Progressive disease	Progressive-stable disease	Complete clinical remission—no evidence of disease

Largest lesion volume (cm^3^)	>13	5–13	<5

Number of lesions	≥3	2	1

**Table:** (c) Graded prognostic assessment (GPA)

	Score 0	Score 0.5	Score 1
Age (years)	>60	50–59	<50

KPS	<70	70–80	90–100

Number of CNS metastases	>3	2-3	1

Extracranial metastases	Present	—	None

**Table 2:** Number of patients according to primary tumor histology and dominant lesion localization.

Histology	Patients
Lung	99
Kidney	22
Bowels	16
Melanoma	14
Breast	12
Liver	2
Prostate	1
Testicle	1
Bladder	1
Ovary	1
Uterus	1
Unknown	34

Total	204

Localizations	Number of main lesions

Frontal	66
Posterior fossa	54
Parietal	35
Temporal	26
Occipital	17
Sella	4
Meninges	2

Total	204

**Table 3:** Average and median survival in patients with brain metastases according to RPA index. Note differences between average and median survival probably due to clinical influencing factors not taken into account.

RPA	Average survival	Median survival
Class I	19.71	10.3

Class II	16.4	8.3

Class III	10.27	3.6

**Table 4:** Average and median survival in patients with brain metastases according to primary tumor histology. Note differences between average and median survival probably due to influencing factors not relating only with histology.

Histology	Average survival	Median survival
Breast	19.01	10.3

Bowels	11.17	11.0

Kidney	32.9	6.5

Lung-NSCLC	19.75	11.15

Lung-SCLC	10.8	10.25

Melanoma	10.83	8.0

Unknown	11.64	3.6
